# Palliative care for persons with Parkinson’s disease: a qualitative study on the experiences of health care professionals

**DOI:** 10.1186/s12904-019-0441-6

**Published:** 2019-07-09

**Authors:** Herma Lennaerts, Maxime Steppe, Marten Munneke, Marjan J. Meinders, Jenny T. van der Steen, Marieke Van den Brand, Dorian van Amelsvoort, Kris Vissers, Bastiaan R. Bloem, Marieke Groot

**Affiliations:** 1 Department of Neurology, Donders Institute for Brain, Cognition and Behaviour, Radboud university medical center, Reinier Postlaan 4, 6500 Nijmegen, AB The Netherlands; 2 Department of Anesthesiology, Pain and Palliative Medicine, Radboud university medical center, Nijmegen, The Netherlands; 3 Department of Primary and Community Care, Radboud university medical center, Nijmegen, The Netherlands; 4 Department of Public Health and Primary Care, Leiden University Medical Center, Leiden, The Netherlands; 5 Dutch Parkinson’s Disease Association, Bunnik, The Netherlands; 6 Scientific Center for Quality of Healthcare, Radboud Institute for Health Sciences, Radboud university medical center, Nijmegen, The Netherlands

**Keywords:** Parkinson’s disease, Palliative care, Barriers, Facilitators, Focus groups, Qualitative approaches

## Abstract

**Background:**

Parkinson’s disease (PD) is a chronic and neurodegenerative disease associated with a wide variety of symptoms. The risk of complications increases with progression of the disease. These complications have a tremendous impact on the quality of life of people with PD. The aim of this study was to examine health care professionals’ experiences of potential barriers and facilitators in providing palliative care for people with PD in the Netherlands.

**Methods:**

This was a qualitative descriptive study. The data were collected from 10 individual in-depth interviews and three focus groups (*n* = 29) with health care professionals. Health care professionals were selected based on a positive answer to the question: “In the past 2 years, did you treat or support a person with PD who subsequently died?” The data were analyzed by thematic text analysis.

**Results:**

Health care professionals supported the development of a palliative care system for PD but needed to better understand the essence of palliative care. In daily practice, they struggled to identify persons’ needs due to interfering PD-specific symptoms such as cognitive decline and communication deficits. Timely addressing the personal preferences for providing palliative care was identified as an important facilitator. Health care professionals acknowledged being aware of their lack of knowledge and of their little competence in managing complex PD. Findings indicate a perceived lack of care continuity, fragmentation of services, time pressure and information discontinuity.

**Conclusions:**

Health care professionals experienced several facilitators and barriers to the provision of palliative care to people with PD. There is a need to improve the knowledge on complex PD and the continuity of information, as well as optimize coordination and deliver care based on a persons’ preferences. Additional training can help to become more knowledgeable and confident.

**Electronic supplementary material:**

The online version of this article (10.1186/s12904-019-0441-6) contains supplementary material, which is available to authorized users.

## Background

Parkinson’s disease (PD) is a chronic, progressive disease for which there is no cure. Affected persons may experience a wide range of symptoms, such as immobility, pain, fatigue, sleeping problems, cognitive deficits or dementia [1–3]. Therefore, professionals from various disciplines are involved in the care for persons with PD and their family caregivers. Because PD progresses slowly, in the early phase it is generally not considered as a terminal illness. But PD does increase mortality, although PD is usually not listed as the immediate cause of death [4, 5]. Most persons with PD die from complications such as dementia, pneumonia, infections or fall-related injuries [5–8]. Furthermore, late-stage PD is associated with considerable suffering, comparable with end-stage cancer [9].

The World Health Organization (WHO) has defined palliative care as an approach that improves quality of life of patients and families facing the problems associated with life-threatening illnesses [10]. Many healthcare professionals from a wide range of professional disciplines can provide “generalist palliative care”. In addition, specialist palliative care (specialised or specialty palliative care) can be considered when the patient’s or family’s needs exceed the competence and confidence of healthcare professionals or when access to certain services is required. The need to further develop and implement specialised palliative care for persons with PD has received more attention over the last decades [11–13]. However, despite these efforts, it is often not a common component of current PD care programs. Furthermore, it is unclear how “generalist palliative care” in PD should be provided. Yet, it is important to initiate palliative care at the appropriate disease stage, preferably in addition to a more disease-oriented approach [12–14].

Health care professionals’ experiences with and perceptions of palliative care in PD are known from a few studies [15–18]. It appears that many professionals lack the necessary competences for palliative care, feel inexperienced and want more education in this field [15–17]. We conducted a large qualitative study based on previous research to gain a more in-depth view on these aspects and add new ones that can help to improve palliative care.

## Methods

### Study design

This qualitative study is part of the ‘ParkinsonSupport–project’ that aims to improve the palliative care for persons with PD and their families in the Netherlands [19]. We conducted individual semi-structured interviews with 10 health care professionals and held three focus group discussions with 29 health care professionals. Recruitment and data collection took place from September 2016 till March 2017.

### Selection of participants

Health care professionals were recruited from ParkinsonNet. This concept is reviewed in detail elsewhere [20, 21]. Briefly, ParkinsonNet is a Dutch professional healthcare network with nationwide coverage, consisting of 70 regional networks which encompass health care professionals specialized in PD (*n* = 3,171) [20, 22, 23]. The central idea is that persons with PD should preferentially be treated by a small group of selected professionals with a high degree of expertise in PD. This expertise is gained initially during a 3-day training course, and is maintained at a high level because patients are being referred specifically to these ParkinsonNet therapists, resulting in high case loads and opportunities to maintain or even increase expertise [24]. Patients in the Netherlands are motivated to seek professional care from members of the ParkinsonNet, but this is not compulsory, and patients are free to also select more generically active professionals. Importantly, recent work showed that patients treated by ParkinsonNet professionals are very comparable to patients treated by generically trained therapists, e.g. in regards to age, gender or education level [25]. The coverage of ParkinsonNet depends on the professional discipline, and varies between around 60% of patients for physiotherapy to around 90% for speech-language therapy (Darweesh et al., unpublished observations).

All health care professionals from ParkinsonNet received an email and those who confirmed they had treated or supported a person with PD in the past 2 years, who subsequently died, were invited to fill in a questionnaire (unpublished data). The final question concerned willingness to elaborate further on the topic in a semi-structured interview or a focus group discussion. Purposive sampling was employed to ensure the sample represented a diverse range of health care professionals and practice characteristics such as age, sex, experience and professional background. Next, we divided health care professionals in two groups, individual interview or focus group discussion. Standard procedures were employed for obtaining informed consent from the professionals who eventually participated in the individual interviews or focus group discussions. Most non-health care professionals declined because of time issues. A detailed description of health care professionals in the interviews and focus group discussions is presented in Table 1 “Characteristics of participants”.

**Table 1:** Characteristics of participants

	Individual interview (*n* = 10)	Focus group discussion (*n* = 29)
Gender		
Women	7	26
Professional background		
Neurologist	1	1
Elderly care physician	1	2
Psychologist	–	1
PD nurse practitioner	1	6
Community nurse	1	1
Physiotherapist	1	10
Speech therapist	1	3
Occupational therapist	1	3
Dietician	1	2
Other^a^	2	–
Age		
25–34	1	2
35–44	4	4
45–54	1	11
55–64	4	11
65+	–	1
Highest educational level		
Secondary education	–	2
Higher education	5	20
University	5	7
Setting		
Academic hospital	3	3
Community hospital	1	3
Nursing home	1	12
Private clinic	2	8
Home care	2	2
Hospice	–	–
Rehabilitation centre	–	2
Primary healthcare centre	1	4

^a^Other: general practitioner & psychiatrist

To develop the interview guide, we first conducted a literature review in PubMed in 2016 using search terms like ‘palliative care’, ‘Parkinson’s disease’ and ‘end of life care’. Our aim was to find relevant studies on persons with PD and (defining) palliative care including expert opinions, case studies and empirical studies [9, 12, 15–18, 26–37]. Furthermore, as indicated earlier, we sent out a questionnaire to gain more information on professionals’ knowledge and competence in palliative care. An interview guide was developed starting from the research gap and questionnaire results (unpublished data). The guide was discussed and amended if needed by HL, MS and MG. An expert panel consisting of three health care professionals and two researchers with experience in either PD, palliative care or both, reviewed this version and, where thought appropriate, adjusted it. It consisted of four topics, each comprising multiple open questions (Additional file 1).

### Data collection

Two of the authors (HL or MS) held individual semi-structured interviews with 10 health care professionals. Each interview took between 60 and 90 min. All interviews took place at professional’s place of work or preferred location, so that the interviewers could get a good feeling with the matters discussed. We conducted three focus group discussions which, on average, took 90 min each. Focus group discussions were organized at a central place in the Netherlands. The sessions were chaired by either MG or MM, assisted by either HL or MS. One served as a moderator who fostered an active and open discussion, and the other served as an assistant who took notes. There was no prior relationship between the researchers and the participants and before the start of the interview, only the interviewers’ names and occupations were mentioned. See Table 2 “Researchers’ characteristics” for more information regarding researchers’ characteristics. The number of health care professionals in the focus groups ranged from eight to ten. The focus group discussions served to engage the participating professionals in considering findings from the individual interviews. Specifically, we used the individual interviews to solicit individual views and experiences. In contrast, the focus group discussions served to engage the professionals in further discussions about similar topics.

**Table 2:** Researchers’ characteristics

Code	Initials	Gender	Age	Occupation and experience
I1	H.L.	Female	35	PhD candidate, master degrees in Social Sciences. 5 years experience on multiple projects for PD, nursing care and 14 years experience in PD-patient care.
I2	M.S.	Female	29	Master degrees in Psychology. Two years experience on multiple projects for PD and 5 years employed as coordinator at ParkinsonNet, a Dutch nationwide PD network
I3	M.G.	Female	51	Senior researcher, assistant professor, PhD in Palliative Care, over 18 years of experience on research projects in palliative care, senior lecturer Qualitative Research, Nurse (NP)
I4	M.M.	Male	50	Associate professor in healthcare innovation, director of strategy Movement Disorders Centre of Expertise, Managing Director ParkinsonNet, 17 years of experience on multiple multidisciplinary research and innovation projects for PD

### Data analysis

All semi-structured interviews and focus group discussions were audio-taped and transcribed. We used an inductive analysis, involving the conceptualization of themes from the transcripts. Two researchers (HL & MS) read and reread the transcripts and coded the first four transcripts using open coding. To increase coding reliability, all transcripts were initially coded by HL and MS separately. Open codes were compared and discussed until an initial code tree was established. All interviews and focus group transcripts were further analysed indepently. Codes were discussed, added, modified or merged if necessary. After coding three interviews and two focus group discussions with the code tree no new codes emerged and data saturation was reached.

Afterwards a process of sorting and classifying codes into subthemes and themes started. When differences in interpretation between researchers remained, a third senior researcher (MG) was consulted. The reliability of the findings was enhanced further by scrutiny from the project group, which included researchers and practicing clinicians from different fields (experiences in palliative care or parkinson care). Once consensus was reached, a final set of themes and subthemes was decided upon [38–40]. The software package Atlas Ti-8 supported the qualitative data analysis [41].

## Results

The analysis resulted in four themes and 13 subthemes (see Table 3 “Perceived facilitators & barriers for palliative care for persons with PD”) relating to perceived barriers and facilitators for palliative care for persons with PD and their families. The themes are described in detail below with supporting data that are presented in Table 4 “Quotes taken from the interview and focus group discussions”. In the discussions we departed from the definition on palliative care (see Additional file 1; interview guide theme 1 ‘defining palliative care in PD’) from this current analysis.

**Table 3:** Perceived facilitators & barriers for palliative care for persons with PD

Themes	Barriers	Facilitators
Addressing needs of persons with PD and family caregivers	A persons cognitive deficits and communication problems Tension between needs from a person and his/her family caregivers A lack of time in interaction with family caregivers	Early speaking about wishes and needs with person, family and health care professionals
Disease management	Lack of clear responsibilities and roles in (introducing) palliative care Limited resources; lack of time, high workloads and financials	More evidence and guidance in offering adequate disease management
Professionals need for training	A lack of competences and specifically for the spiritual domain	Training helps in feeling more confident Communication skills; an open and sensitive attitude
Connection between services	Limited communication between health care professionals	Availability of specialized palliative care services Care coordination; need for a central figure to coordinate palliative care

**Table 4:** Quotes taken from the interviews and focus group discussions

Quote number	
Q1 Theme: Addressing needs of persons with PD and their family caregivers	This female with PD is in a phase that we need to make choices for the future. We do not know what she wants and how she think of her end of life…. Now, she has several cognitive problems… shouldn’t these conversations have been introduced earlier in the trajectory when this women was cognitively at a better level? (individual interview, nurse practitioner) Many people with PD at advanced stage can’t communicate anymore. They can live their life but we can’t communicate on that moment about what they want and what is important for them. (focus group discussion)
Q2 Theme: Addressing needs of persons with PD and their family caregivers	People will stay focused on something like hope. Maybe there are still opportunities that will help them. They try, have new hopes, but also are often disappointed. (focus group interview, occupational therapist) Speaking about prognosis should be done earlier. You see that if a person goes to a nursing home (...) they often expect that the condition will stabilize. But PD will continue and you need to discuss scenarios before things get worse. (focus group discussion, elderly care physician)
Q3 Theme: Addressing needs of persons with PD and their family caregivers	What I find really difficult is when a family caregiver is too overburdened and puts a patient in a wheelchair for the whole day. As a caregiver said: “At least he doesn’t stand in the way then and cannot fall. The more immobile my husband is the better, that’s easier for me. But it is very emotionally taxing. (focus group discussion, occupational therapist) When a patient became ill, his partner pushed for a hospital admission. I don’t think that it was what he wanted, or what maybe was best for him. But this partner, she wanted to do something… (focus group discussion, physiotherapist)
Q4 Theme: Disease management	When you get diagnosed with PD, you will in fact fight against the disease. You form an alliance for many years with your specialist too fight for a better quality of life or to remain stable. It is very hard for a specialist to say, *I cannot do anything more for you, I have to quit this fight*. Telling this to a patient is tough. (individual interview, elderly care physician) Saying that a patient is in his last month of his life, I think you get different interventions, focusing more on comfort than on treatment. I think we (professionals) feel and think about it, but don’t explicitly say it. (focus group discussion)
Q5 Theme: Disease management	You can have all kind of side effects through increasing the medication doses. For example, clozapine therapy carries a high risk of dizziness and balance problems. Balancing between pros and cons is very difficult. (focus group discussion, elderly care physician) Parkinson medication is troublesome. It’s hard to judge whether medication is effective or not. Should we give more medication or should we give less medication instead to reduce side effects? Or should we quit trying to improve balance in medication completely?” (focus group discussion, elderly care physician)
Q6 Theme: Professionals need for training	I would never mention the word palliative to a patient. No, that’s not my thing. (individual interview, occupational therapist) You have to get used to speak about difficult issues. However, you need to persuade yourself to do this and it will eventually get easier. (individual interview, nurse practitioner)
Q7 Theme: Professionals need for training	I think doctors won’t speak in depth about spirituality with their patients. Medical treatment is, of course, our core business and this (*spirituality)* often is not a subject. I hope also that our PD-nurse pays attention to this. (individual interview, neurologist)
Q8 Theme: Professionals need for training	Well, I won’t speak about dying if I see a patient for only a short time. But when I build a relationship with a patient, it is different. Even when a patient is physically stable, I probe whether they had ever thought of when the situation gets worse. (individual interview, physiotherapist) A personal relation and connection with a patient is important; sometimes it is a nurse, a physiotherapist, or a cleaner who has known a person for a couple of years. (individual interview, occupational therapist)
Q9 Theme: Connection between services	I am also a member of a palliative care team in our region. The number of referrals for persons with PD is depressingly low. I think that should be different… I know there are many cases and why do they not consult us? (individual interview, elderly care physician)
Q10 Theme: Connection between services	What I often hear, is that there is a need for better coordination by for example a case manager. Especially when a patient comes from a situation of transfers between hospital, rehabilitation centre and home. There are changes in medication regimen, feeding or therapy from a physiotherapist. People do not always know what is agreed upon (focus group discussion)

### Addressing needs of persons with PD and family caregivers

One of the key issues in late-stage PD is that the person’s needs and wishes can be difficult to elicit due to communication problems and/or cognitive decline (Table 4, Q1). A number of respondents cited that not speaking timely about needs might complicate treatment. In some cases, when the person with PD could not understand the purpose of a proposed treatment, it was hard to tell whether the treatment might be too burdensome. Health care professionals mentioned that they were not certain about what persons with PD themselves want. Early discussions about wishes was seen as a facilitator for improving palliative care (Table 4, Q2). A barrier that was mentioned by a few HCP was that a PD trajectory is less predictable than for example for cancer. Persons with PD might be focusing on stabilizing instead of their general decline (Table 4, Q2). HCP emphasized the urgency of timely speaking about wishes and needs (due PD specific symptoms) as it might enable them to provide future care that is based on a person’s needs. However, ‘timely’ was not well defined. On the other hand, HCP argued that in some cases persons were not ‘open’ for having these conversations.

According to health care professionals, involvement of family caregivers in the care for PD was seen as a facilitator, but also as a barrier. In situations where a person cannot clarify needs or make decisions, health care professionals intensified collaboration with family caregivers so as to be able to offer care in accordance with the person’s needs. However, a few health care professionals recognized that people with PD and caregivers may have different needs, in which case it is difficult to determine whose needs must be prioritized (Table 4, Q3). Health care professionals also noted that emotionally burdened family caregivers can hinder persons’ adjustment to their disease (Table 4, Q3). Care for family caregivers was seen as an important, but also as a complicating factor. Furthermore, many professionals experienced a lack of time for interaction with family caregivers and/or bereavement support.

### Disease management

The theme disease management occurred on a meso- and a micro level. On the meso level, health care professionals were unsure of the timing and introduction of palliative care into the care pathway and by whom. Many healthcare professionals noticed that palliative care should be the role and responsibility of a physician. There was a lack of role descriptions in providing palliative care and a need to clarify the roles and responsibilities of different healthcare professionals.

Health care professionals mentioned that in case of neurologists, they often lose track of their patients when these become too frail to attend an outpatient clinic. Another probable reason for not discussing palliative care is the longstanding relationship between a neurologist and a person with PD with its focus on optimizing medical treatment to suppress symptoms. If suppressing symptoms is not feasible anymore, it might be painful for a person with PD to accept that drug treatment is less effective and for doctors to verbalize ‘bad news’ (Table 4, Q4).

On micro level, many health care professionals mentioned a lack of evidence about interventions as a barrier in offering adequate symptom management. Physicians’ brought forward that adjusting medication becomes extremely difficult when PD progresses. Few medication treatment goals remain and often the therapeutic effect aimed for does not weigh against the occurring side-effects.(Table 4, Q5) Furthermore, occupational and physical therapists specifically mentioned the need for more knowledge on how to support persons with PD in day-time tasks, comfortable sitting and lying, or prevention from pressure ulcers. Another topic, mentioned by physicians, dieticians and speech therapists, was the placement of a feeding tube as a life-prolonging intervention. If a feeding tube was already in place, deciding whether to leave it in place or to remove it was perceived as difficult. Ethical issues concerning boundaries between curative care and palliative care as well as possible legal implications of end-of-life decisions are not clear. Although health care professionals realized that to provide patient centered care achievable, tailored interventions are needed. However, more general guidance and evidence are wished for disease management in PD.

Overall, many health care professionals reported barriers to the provision of palliative care such as high workloads, a lack of time and restrained financial resources.

### Professionals need for training

A range of experiences from clinical practice in relation to limited competences and skills in working with persons with PD were described. Health care professionals found themselves at the stage of ‘conscious incompetence’ [42]. They recognized the deficit of not knowing how to offer palliative care or not knowing what skills are needed. It was suggested that additional training can help overcome these deficits. Training could also help in feeling more confident in using the knowledge and skills. But also in helping health care professionals to address issues related to death and dying. Some professionals felt reluctant because of their own personal issues, such as taboo, uncertainty or personal life experiences and beliefs. (Table 4, Q6).

Spiritual care was much less actively discussed among health care professionals than the other domains of palliative care (physical, social, psychological). A few health care professionals remarked that spirituality was addressed only if the person with PD explicitly raised spiritual issues and was not always a standard subject in care. (Table 4, Q7) When professionals were confronted with spiritual care, they frequently referred to or involved others. Health care professionals underpinned spiritual care as an important part of palliative care. However, the awareness for spiritual needs and how to respond or act as a professional remains unclear. Some suggested that it was not enough to develop training on this topic.

Many health care professionals noted that ‘good communication skills’ is a facilitator. Communication was also connected with personal attitude. An open and sensitive attitude to persons with PD might help them to speak freely and honestly about expectations and fears. Talking about difficult issues was easier if trust had been established between a health care professionals and a person with PD. A longstanding or intensive patient-professional relation was another facilitator mentioned. (Table 4, Q8).

### Connection between services

The availability of specialist palliative care (SPC) services in a hospital was seen as a facilitator to improve PD care. However, some professionals had not heard from SPC services before and other health care professionals did not know how SPC could contribute in late-stage PD. Only a small group had often contact with a SPC services. An elderly care physician who was also working in an SPC service, said he hardly saw persons with PD and his expertise could be used more often. (Table 4, Q9) A further integration of SPC services could help the organization of palliative care for people with PD. Furthermore, a number of health care professionals identified a lack of communication and information continuity in situations where multiple health care professionals were involved. More multidisciplinary communication could also help in using each specific knowledge in providing palliative care. Furthermore, health care professionals highlighted the need for a central figure in palliative care for PD. Although many participant agreed that the general practitioner could be this person, there was some questioning of the appropriateness and involvement of GP’s in PD. Health care professionals cited barriers, such as their lack of PD knowledge, lack of time for care coordination and limited accessibility for other professionals. Other health care professionals pointed out that a case manager liaising with other professionals might be a facilitator to improve and ensure continuity of palliative care. (Table 4, Q10). A few professionals mentioned that a casemanager could be ‘a new person’, while others saw a casemanager more as an approach supporting teamwork, common goals, and a willingness to involve whoever had the appropriate skills.

## Discussion

This study provides insight into how a variety of health care professionals experienced palliative care provision for persons with PD. Health care professionals find it hard to identify needs of persons with late-stage PD due to impaired communication and declining cognitive functioning. Therefore, speaking timely on a persons’ wishes and needs for future care can help them to prepare for what might come. As a result, health care professionals are more able at the end of life to provide patient centered care. It also appeared that working in partnership with family caregivers becomes more crucial in late-stage PD, although this may not be easy. The needs of persons with PD and caregivers may not always be congruent and family dynamics may hinder the provision of optimal support [43, 44]. Persons with PD, family caregivers and health care professionals need to work together to plan care based on a person’s wishes and needs. From studies on the perspectives of persons with PD and caregivers, it becomes clear that preferences on when to discuss end-of-life issues vary and that only half of the persons are ready to discuss advanced care documents in an early stage of the disease [45]. One of the reasons for this hesitant attitude is the lack of information and support from health care services [31, 35]. From the health care professionals’ perspective, however, early planning, before a person with PD loses communication capacity, is preferable for providing optimal palliative care [16, 17, 46].

We found a need for further education about “general palliative care” for health care professionals. Additional training was seen as a facilitator for health care professionals to become more competent and confident. Two studies have reported similar findings regarding competences and knowledge [15–17]. Improving one’s knowledge about palliative care can help health care professionals overcome barriers in palliative care [47, 48] Special attention may be paid to spirituality as also many other health care professionals struggle with the concept of spirituality in relationships with patients, caregivers and themselves [49].

The interviewed health care professionals were positive about collaboration with SPC services, although such collaborations were still rare in daily practice. Concrete examples of how this collaboration could be realized are needed. Earlier studies [16, 17] also found that PD care should include a collaboration with an SPC service so as to be able to meet the palliative care needs of persons with PD. Patients who received care from an SPC service were more satisfied about care [50]. Health care professionals overall were not well informed about whom to refer to and when, and they were uncertain about when to initiate palliative care [16, 17, 51]. This can lead to inappropriate or no referral at all, and persons with PD missing the benefits of a palliative care approach. In the literature, different models of improving palliative care have been proposed; this includes consultative palliative care teams, integrated palliative care programs and complementary models (including primary palliative care, mobile consultation team, an acute palliative care unit, and an outpatient supportive care clinic) [11–13, 52, 53]. Although different models can help to optimize palliative care in PD, further research is needed on outcomes of these various models in daily practice.

## Strengths and limitations

The use of multiple qualitative methods (individual interviews and focus groups discussions) and investigator triangulation (data coding by more than one person) ensured the validity of our approach. A particular strength of this study is that it inventoried the experiences of a wide range of health care professionals from several settings. This allowed us to gather a broad view of palliative care for PD. As a limitation, we did not included health care professionals with no or short experience in palliative care. The participating health care professionals might have been keener to have a good palliative care system because they have a recent experience with a person with PD who died in the past 2 years. Consequently these findings do not reflect views from more inexperienced health care professionals. A further limitation is that our data present health care professionals’ reported practice, we did not quantitatively measure the health care professionals’ knowledge and experiences. Lastly, our findings represent the situation in the Netherlands and may not generalize to a wider international context. Despite these limitations, the findings of this study do point to ways of improving the quality of palliative care for persons with PD.

## Future perspectives / clinical relevance

Early identifying needs and discussing the preferences of a person with PD should become a part of PD care. Furthermore, educational strategies are needed to increase health care professionals’ knowledge of palliative care. Appropriate strategies include workshops, written material and online learning modules. Based on the outcomes of this study, we developed an information film for persons with PD and their families (only available in Dutch: https://www.youtube.com/watch?v=8W02j6fzd3g). Collaboration with an SPC service can help to learn on a case-by-case basis. Competence gaps can have implications for care as persons with PD may experience significant delay in symptom control, until they are referred to appropriate specialist services. Measures to increase health care professionals’ competence should be implemented to improve quality of care. Obviously, it would be relevant to gain more insight into the experiences of persons with PD themselves at advanced disease stages [54]. Exploring this is notoriously difficult due to communication and cognitive deficits that increase with advancing PD.

## Conclusion

Our results has identified several barriers and facilitators in providing palliative care for people with PD. Due to specific PD-symptoms there is a need of an early proactive approach to identify palliative care needs. Furthermore, health care professionals experienced a need to improve their knowledge and skills in palliative care.

## Additional file

Additional file 1: Interview guide for individual interviews and focus group discussions. (DOCX 39 kb)

## Data Availability

The datasets used and/or analyzed during the current study are available from the corresponding author on reasonable request.

## Abbreviations

COREQ: Consolidated criteria for reporting qualitative research
GP: General Practitioner
PD: Parkinson’s Disease
SPC: Specialist Palliative Care
WHO: World Health Organization
